# miR-425-5p Regulates Proliferation of Bovine Mammary Epithelial Cells by Targeting *TOB2*

**DOI:** 10.3390/genes15020174

**Published:** 2024-01-28

**Authors:** Yuchao Li, Guanhe Chen, Shuxiang Xu, Siqi Xia, Wenqiang Sun, Jie Wang, Shiyi Chen, Songjia Lai, Xianbo Jia

**Affiliations:** 1 Farm Animal Genetic Resources Exploration and Innovation Key Laboratory of Sichuan Province, Sichuan Agricultural University, Ya’an 625014, China; lichao_1116@163.com; 2 Key Laboratory of Livestock and Poultry Multi-Omics, Ministry of Agriculture and Rural Affairs, College of Animal Science and Technology, Sichuan Agricultural University, Ya’an 625014, China; cgh_0132@163.com (G.C.); 17780544390@163.com (S.X.); xiasiqi2020@163.com (S.X.); wqsun2021@163.com (W.S.); wjie68@163.com (J.W.); chensysau@163.com (S.C.); laisj5794@sicau.edu.cn (S.L.)

**Keywords:** mammary epithelial cells, miR-425-5p/*TOB2* axis, cellular proliferation, heat stress

## Abstract

**Simple Summary:**

The substantial impact of heat stress on the milk yield of dairy cows necessitates improving the physiological condition of their mammary glands to enhance dairy production efficiency. The present study observed that heat stress exerted a suppressive effect on the proliferation of bovine mammary epithelial cells (BMECs) and significantly downregulated the expression of endogenous miR-425-5p. The experimental findings confirmed that overexpression of miR-425-5p promotes the proliferation of BMECs. It was identified that Transducer of ERBB2, 2 (*TOB2*) is a target gene of miR-425-5p. Overexpression of *TOB2* inhibited the proliferative effect of miR-425-5p on BMECs. These results demonstrate that miR-425-5p promotes the proliferation of BMECs by targeting *TOB2*. These findings offer a dependable reference for addressing the issue of reduced milk production in dairy cows experiencing heat stress at the molecular level.

**Abstract:**

In recent years, rising temperatures have caused heat stress (HS), which has had a significant impact on livestock production and growth, presenting considerable challenges to the agricultural industry. Research has shown that miR-425-5p regulates cellular proliferation in organisms. However, the specific role of miR-425-5p in bovine mammary epithelial cells (BMECs) remains to be determined. The aim of this study was to investigate the potential of miR-425-5p in alleviating the HS-induced proliferation stagnation in BMECs. The results showed that the expression of miR-425-5p significantly decreased when BMEC were exposed to HS. However, the overexpression of miR-425-5p effectively alleviated the inhibitory effect of HS on BMEC proliferation. Furthermore, RNA sequencing analysis revealed 753 differentially expressed genes (DEGs), comprising 361 upregulated and 392 downregulated genes. Some of these genes were associated with proliferation and thermogenesis through enrichment analyses. Further experimentation revealed that *TOB2*, which acts as a target gene of miR-425-5p, is involved in the regulatory mechanism of BMEC proliferation. In summary, this study suggests that miR-425-5p can promote the proliferation of BMECs by regulating *TOB2*. The miR-425-5p/*TOB2* axis may represent a potential pathway through which miR-425-5p ameliorates the proliferation stagnation of BMECs induced by HS.

## 1. Introduction

The cow mammary gland was a vital reproductive organ composed of various cell types, including mammary epithelial cells, adipocytes, fibroblasts, and immune cells. Among these, mammary epithelial cells (MEC) possess a secretory function and thus play a pivotal role in the milk production of cows [1]. MECs consist of two layers: luminal cells, responsible for milk synthesis, and basal myoepithelial cells, which assist in milk ejection. Mammary epithelial cells were the fundamental units responsible for synthesising and secreting milk in the mammary gland. They also play a crucial role in immune protection against external pathogens [2].

Heat stress was the collection of non-specific physiological responses exhibited by an organism in a high-temperature environment [3]. Previous research has shown that heat stress in animals mainly results in oxidative stress, disrupted hormone secretion, imbalanced cytokines, cellular autophagy, and abnormal cell functions [4]. In high-temperature conditions, elevated body temperature, respiratory rate, and heart rate in cows can affect feed intake and productivity [5,6]. In addition, the elevated temperatures can lead to a decline in both milk yield and quality in cows [7]. Heat stress also affects the production of reproductive cells in both female and male animals, leading to a significant reduction in reproductive performance in cows [8]. Moreover, a prolonged heat stress in livestock has been shown to reduce immune function, making them more vulnerable to infectious and metabolic diseases [9].

The microRNAs (miRNAs) were a group of endogenously encoded RNAs that consist of approximately 18 to 25 nucleotides in length [10,11]. They were conserved across species and exhibit temporal and tissue-specific expression patterns [12]. MiRNAs can regulate the expression of thousands of genes in normal physiological and pathological conditions. The degree of complementarity between miRNA and its target genes determines the basis for the selection of silencing regulatory mechanisms. For example, when miRNA and target gene mRNA exhibit almost complete complementarity, it leads to mRNA degradation. These regulatory mechanisms were frequently observed in plants and fungi [13]. In contrast, in mammals, when the 3′UTR of the target gene only partially complements with the seed sequence of miRNA, it results in the translational inhibition of mRNA [14,15]. In most studies, it has been demonstrated that miRNAs bind to target mRNAs and function as regulators by suppressing the expression of the latter. However, some studies suggest that miRNAs can interact with the promoter or 5′UTR of target mRNAs, which may promote gene expression instead of inhibiting it [16,17,18].

The transducer of ERBB2 (*TOB2*) was a member of the mammalian anti-proliferation protein family, with a primary function of inhibiting cell proliferation. The family comprises six members, including *BTG1*, *BTG2*, *BTG3*, *BTG4*, *TOB1*, and *TOB2* [19]. Previous studies have shown that *TOB2* can participate in and regulate the cell cycle of a wide range of cells [20,21]. However, the mechanism of *TOB2* function in BMECs is still under-researched.

MiR-425-5p is a miRNA that regulates the expression of target mRNAs, affecting the cell cycle. It has been implicated in cancer cell migration, proliferation, and apoptosis in cancer research [22,23] and identified as a biomarker in metastatic prostate cancer [24]. Additionally, miR-425-5p inhibits the differentiation and proliferation of preadipocytes in pig muscle [25]. Previous studies have postulated that miR-425-5p may exert a regulatory effect on cellular value-addition; however, no investigations have been conducted thus far to elucidate the role of miR-425-5p in BMEC. Therefore, this study aimed to investigate whether miR-425-5p could mitigate the stagnation of value-addition in BMEC caused by HS and explore its underlying mechanism.

## 2. Materials and Methods

### 2.1. Cell Culture

The BMEC cells utilized in this study were procured from CELLBIO, a reputable supplier based in Shanghai, China. Similarly, the HEK-293 cell line was obtained from the Cell Resource Centre of the Shanghai Institutes for Life Sciences, China. The subsequent cellular cultures were maintained in Dulbecco’s Modified Eagle’s Medium (DMEM) supplemented with 10% Fetal Bovine Serum (FBS), 100 U/mL penicillin, and 0.1 mg/mL streptomycin under incubation conditions of 37 °C with 5% CO_2_ in a humidified cell culture incubator. For subsequent experiments, cells were seeded in 6-well, 12-well, 24-well and 96-well plates (NEST, Wuxi, China, products: 703002, 712002, 702002, and 701002) for experimental manipulations.

### 2.2. HS Models

The HS model was constructed and provided by a senior researcher in our research group [26]. The cell incubation conditions for HS were set at 42 °C (HS), while the control group was kept at 37 °C. The choice of incubating cells at 42 °C was made to simulate severe HS, considering that cattle prefer cold environments over hot. The core body temperature of cattle was typically maintained at 39–40 °C, and HS was most likely to occur when ambient temperatures exceed 40 °C [27].

### 2.3. Transfection

For the transfection of miR-425-4p mimic and inhibitor, liposome transfection reagent (Lipofectamine™ 3000, ThermoFisher Scientific, Waltham, MA, USA) was used to transfect the culture medium, according to the manufacturer’s instructions. Briefly, BMECs were seeded in 96-well, 24-well, 12-well, or 6-well plates, and when the cell density reached 50–60%, the miR-425-5p mimic, miR-425-5p inhibitor, miR-425-5p negative control (NC), and miR-425-5p inhibitor negative control (INC) were transfected for 6 h, after which the medium was replaced with normal growth medium. The subsequently transfected cells can be used for further experimental analysis.

### 2.4. Biological Analysis of Sequencing Data

RNA samples were extracted and assessed for integrity and quality using an Agilent 2100 Bioanalyzer (Agilent Technologies, Santa Clara, CA, USA). The mRNAs containing polyA tails were enriched using oligo(dT) magnetic beads. These mRNAs were then randomly fragmented using divalent cations in NEB fragmentation buffer. Libraries were constructed using either the NEB common library construction method or the strand-specific library construction method. Following quality control, libraries were pooled based on their effective concentration and intended downstream data volume. Illumina sequencing was then carried out. The resulting sequencing data were analysed using the DESeq2 method with a significance level of *p* ≤ 0.05 for differential expression analysis.

### 2.5. Cell Counting Kit-8 (CCK-8) Detection of Cell Viability

Cells were seeded in 96-well plates (NEST Biotechnology, Wuxi, China) at a density of 5 × 10^4^ cells per well. The cells were then transfected with miR-425-5p mimic, miR-425-5p negative control (NC), miR-425-5p inhibitor, and miR-425-5p inhibitor negative control (INC) when the cell density reached approximately 50–60%. CCK-8 solution (10 μL) was added to each well at 0, 12, 24, 36, and 48 h after transfection, according to the manufacturer’s instructions. Cells were then incubated for 2 h. The optical density (OD) values of each sample well were measured at 450 nm. The OD values obtained were subsequently subjected to analysis and visualization using the GraphPad Prism 9 software (GraphPad Software Inc., La Jolla, CA, USA). The OD values at 450 nm were directly correlated with cell viability, thus providing supplementary information regarding the rate of cellular value enhancement.

### 2.6. EDU Assay to Detect Cell Proliferation Efficiency

BMECs were seeded in 24-well plates. After 24 h of transfection, the cells were incubated for 2 h in complete medium supplemented with 50 μL of 5-ethynyl-2′-deoxyuridine (EDU, RiboBio, Guangzhou, China). Cells were then fixed and stained according to the manufacturer’s instructions. The nucleus was bound by DAPI to induce fluorescence, while EDU binds specifically to replicating DNA fragments, resulting in a fluorescent signal in proliferating cells. Following the completion of the staining procedure, the samples were examined using a fluorescence microscope (Olympus, Tokyo, Japan), and images capturing both DAPI and EDU staining within the same field of view were captured. The images of the counted cells were analysed using Image-Pro Plus 6.0 software (Media Cybernetics, Inc., Rockville, MD, USA).

### 2.7. RNA Extraction and Real-Time Fluorescence Quantitative PCR (RT-qPCR)

The kit (Solarbio, Beijing, China) was used to extract total RNA from cell samples, according to the manufacturer’s instructions. We also collected other tissue samples from the bodies of three dairy cows, including the heart, liver, spleen, lungs, mammary glands, back muscles, and skin. The tissue samples were pulverised with liquid nitrogen prior to the extraction of total RNA using the kit. HiScript III RT SuperMix for qPCR (+gDNA wiper) (Vazyme, Nanjing, China) was used for the reverse transcription of mRNA and miRNA. For miRNA, the miRNA 1st Strand cDNA Synthesis Kit (by tailing A) (Vazyme, Nanjing, China) was used. To ensure the accuracy of the subsequent experiments, we assessed the concentration and quality of total RNA using a NanoDrop 2000 spectrophotometer (Thermo Fisher Scientific, San Jose, CA, USA).

RT-qPCR was performed in triplicate on a CFX96 instrument (Bio-Rad, Hercules, CA, USA) using ChamO SYBR qPCR Master Mix (Vazyme, Nanjing, China). The RT-qPCR protocol begins with an initial denaturation step at 95 °C for 3 min, followed by 40 cycles consisting of a denaturation step at 95 °C for 10 s, annealing at 60 °C for 20 s, and extension at 72 °C for 30 s. The levels of mRNA and miRNA were quantified using the 2^−ΔΔCt^ method, with u6 and β-actin as internal references for miRNA and mRNA quantification, respectively.

### 2.8. Western Blotting

The ProteinExt^®^Mammalian Total Protein Extraction Kit (TransGen Biotech, Beijing, China) was used to collect the total intracellular protein, according to the manufacturer’s protocol. The concentration of proteins was determined using the Bradford Protein Assay Kit (Novoprotein, Shanghai, China). The proteins were then separated on a 10% SDS-PAGE gel and transferred to a PVDF membrane. The membrane was transferred and then incubated in 5% skimmed-milk powder for 2 h to prevent non-specific binding. The membrane was then incubated with the appropriate primary antibody at 4 °C for 8 h. After antibody incubation, the membrane was washed three times with TBST and then incubated with a secondary antibody (goat anti-rabbit IgG H&L (HRP), Abclonal, Woburn, MA, USA) for 2 h. The strips were washed three times with TBST solution. Finally, the colour development was performed on an e-BLOT Life Science Instrument Touch Imager Pro (Shanghai, China) using TCL’s Ultra Sensitive Color Developer. The antibodies utilized in this study were PCNA (proliferating cell nuclear antigen) and CDK4 (cyclin dependent kinase 4), which were procured from Zen Bioscience located in Chengdu, China. A secondary antibody purchased from Wuhan Aibotec Biotechnology Co (ABclonal Technology Co., Ltd., Woburn, MA, USA) was used in the experiment.

### 2.9. Dual Luciferase Reporter Gene Assay

The Targetscan database (https://www.targetscan.org/vert_80/, accessed on 21 December 2022) was used to predict the potential target genes regulated by miR-425-5p and to identify the putative binding sites between miRNAs and mRNAs. According to the analysis, *TOB2* was identified as a potential target gene for miR-425-5p. To investigate the interaction between miR-425-5p and *TOB2*, luciferase reporter plasmids were prepared by Tsingke Bio-tech Co., Ltd. (Beijing, China) containing both WT (wild-type) and MUT (mutant) target sequences.

Subsequently, 293T cells were then seeded in 24-well plates. When the cell fusion reached 60%, miR-425-5p mimic or negative control (NC) was co-transfected with wild-type (WT) or mutant (MUT) plasmids into 293T cells, following the transfection steps described above. After 24 h of transfection, luciferase activity was quantified using the Duo-LiteTM luciferase assay system (Vazyme, Nanjing, China).

### 2.10. Results Statistical Analysis of Data

The statistical analysis of the data obtained after the experiment was conducted using the SPSS 26.0 software. Descriptive statistics were performed, and the results were expressed as mean ± standard error of the mean (SEM). The significance of differences between groups was determined using Student *t*-test and one-way analysis of variance (* *p* < 0.05).

## 3. Results

### 3.1. miR-425-5p Is Associated with Breast Development

**Figure 1:**
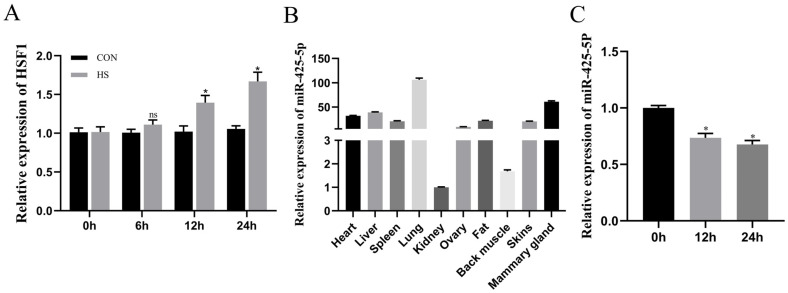
(**A**) The relative expression levels of HSF1 were assessed following the incubation of BMEC in a 42 °C incubator for 0, 6, 12, and 24 h (*n* = 9). (**B**) The expression level of miR-425-5p was determined in different tissues of cows (*n* = 9). (**C**) Changes in miR-425-5p expression levels were measured in BMEC after incubation for 0, 12, and 24 h at a temperature of 42 °C. The data are presented as means ± SEM. * *p* < 0.05, ns (no significance).

As the culture time of BMECs under heat stress conditions increased, the expression levels of heat stress marker genes also increased (*p* < 0.05), confirming the successful treatment of BMECs with heat stress (Figure 1A). The tissue samples of the heart, liver, spleen, lung, kidney, ovary, muscle, fat, skin, and mammary gland of dairy cows were collected, and the expression of miR-425-5p in these tissues was quantitatively verified using real-time fluorescent quantitative RT-qPCR. The results showed that miR-425-5p was highly expressed in mammary gland tissue, significantly different from other tissues (Figure 1B), suggesting a potential association between miR-425-5p and mammary gland development. We also observed changes in the expression level of miR-425-5p under heat stress conditions, showing a significant decrease with prolonged culture time (*p* < 0.05) (Figure 1C).

### 3.2. The Investigation of the Functionality by Differentially Expressed Genes in Response to miR-425-5p

To further investigate the function of miR-425-5p in BMEC, we sequenced BMEC transfected for 24 h. The sequencing data showed 4 million clean reads for each sample, with GC content ranging from 52.28% to 52.44%, with an average content of 52.36%. The reads were meticulously aligned to the Bos taurus reference genome, achieving alignment rates ranging from 96.54% to 97.10% (Table S1). These results indicate high-quality libraries suitable for further analysis.

**Figure 2:**
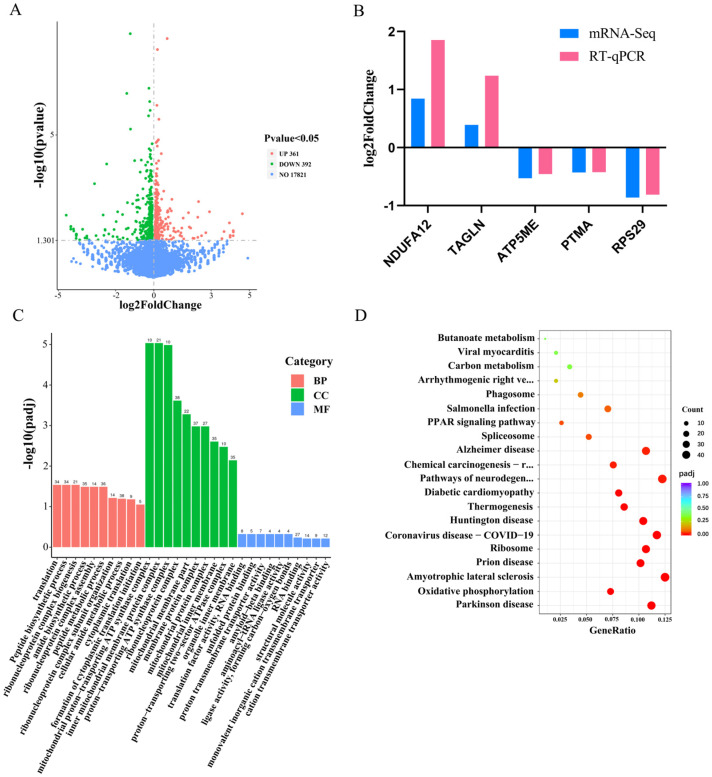
The differentially expressed genes identified through mRNA sequencing assay were experimentally validated and subsequently subjected to comprehensive bioinformatic analyses. (**A**) Volcano plot was generated to visually represent the differential expression of genes meeting statistical significance criteria (padj < 0.05; |log2foldchange| ≥ 0). (**B**) Validation of gene expression changes using RT-qPCR was performed for a subset of five randomly selected differentially expressed genes (*n* = 9). (**C**) Gene Ontology (GO) enrichment analysis focused exclusively on biological process (BP), cellular component (CC), and molecular function (MF), identifying terms significantly associated with the differentially expressed genes. (**D**) Kyoto Encyclopedia of Genes and Genomes (KEGG) pathway analysis revealed the significant enrichment of specific pathways within the lists of differentially expressed genes.

Using DESeq2, 753 differentially expressed genes (DEGs) were identified (Figure 2A) (Table S2). Among them, 361 were upregulated (e.g., ANAPC11, CDKN1A, SMC3, PLK1, YWHAZ, E2F4), and 392 were downregulated (e.g., ROMO1, MT2A, FOXP4). Notably, genes such as ROMO1, MT2A, FOXP4, ANAPC11, CDKN1A, and SMC3 were known to play critical roles in regulating cell proliferation. To validate the sequencing trends, five DEGs were randomly selected for RT-qPCR, which showed consistent results with the sequencing data (Figure 2B). To gain further insight into the role of DEGs, we also performed Gene Ontology (GO) [28] and Kyoto Encyclopedia of Genes and Genomes (KEGG) [29] enrichment analyses. The 753 DEGs were enriched in 2606 GO items, including 1967 biological processes (BP), 335 cellular compositions (CC), and 304 molecular functions (MF) (Figure 2C) (Table S3). The most prominent biological processes included mitotic cell cycle, the regulation of cytokinesis, and ribosomal small subunit biogenesis. Furthermore, KEGG analysis revealed the enrichment of 287 pathways, mainly associated with various diseases and intracellular substance synthesis pathways (Figure 2D) (Table S4). Notable pathways included the IL-17 signalling pathway, oocyte meiosis, p53 signalling pathway, and cell cycle [30,31], which have been implicated in cell cycle regulation.

### 3.3. miR-425-5p Promotes Proliferation of BMECs

**Figure 3:**
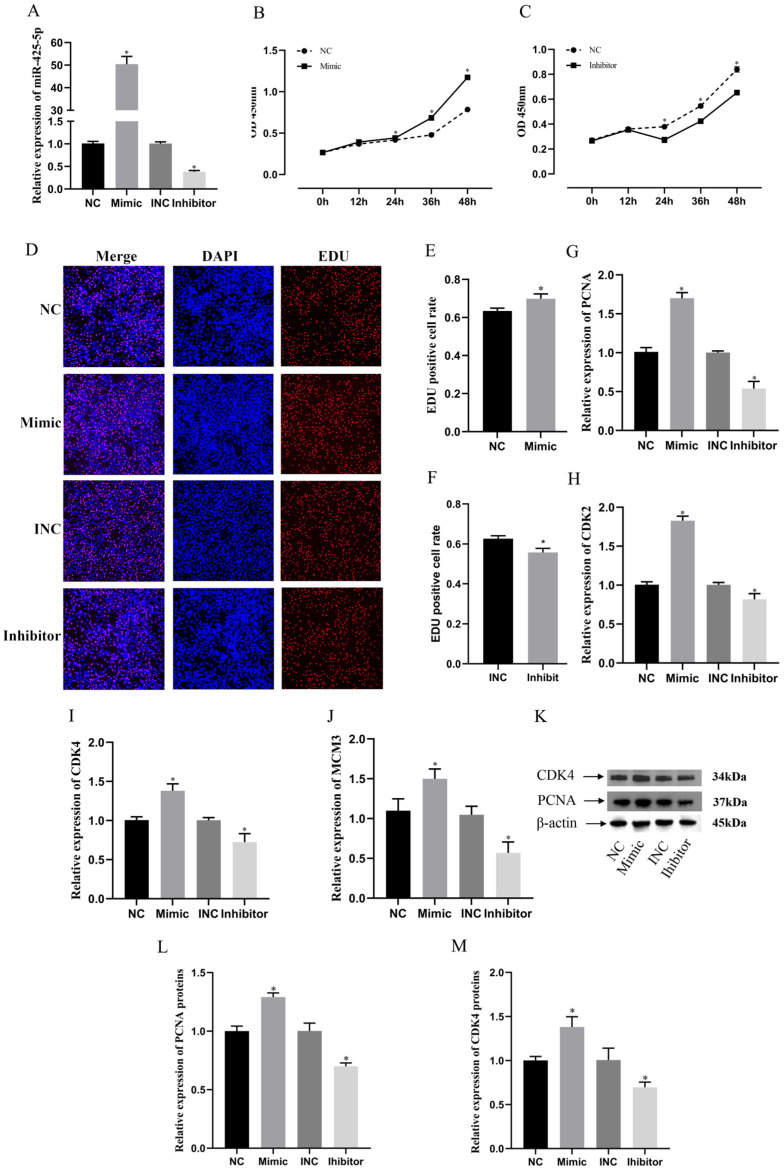
To investigate the impact of alterations in miR-425-5p expression levels on BMEC. (**A**) After overexpression or suppression of miR-425-5p, its relative expression was detected at 24 h (*n* = 9). (**B**,**C**) We assessed changes in BMEC viability following transfection with a miR-425-5p mimic and inhibitor using CCK-8 reagent (*n* = 5). (**D**–**F**) We determined the proportion of proliferating cells in each group using EDU reagent (*n* = 5). (**G**–**J**) We examined the mRNA expression levels of PCNA, CDK2, CDK4, and MCM3 in BMEC after overexpression or inhibition through RT-qPCR analysis with β-actin as an internal control (*n* = 9). (**K**–**M**) protein expression changes in *CDK4* and *PCNA* were evaluated with Western blotting and visualized through their respective grey scale values (*n* = 3). β-actin was used as a reference. The data are presented as means ± SEM. * *p* < 0.05.

To determine the effect of miR-425-5p on BMECs, we examined the expression of miR-425-5p after transfection with mimics or inhibitors. After 24 h of transfection, the cells were harvested, and miRNAs were extracted and reverse-transcribed. Subsequently, the expression level of miR-425-5p was quantitatively determined using RT-qPCR. The results showed that miR-425-5p mimic increased the expression level of miR-425-5p by approximately 50-fold compared to the NC group, whereas miR-425-5p inhibitor decreased the expression level by approximately 0.7-fold compared to the INC group (*p* < 0.05) (Figure 3A). To assess the impact of alterations in miR-425-5p expression on the viability of BMEC, we conducted a CCK-8 assay. According to the CCK-8 results, BMEC cells transfected with miR-425-5p mimic exhibited a significant increase in absorbance at 450 nm after 24 h compared to the NC group. Conversely, the miR-425-5p inhibitor group demonstrated a significant decrease in absorbance after 24 h (*p* < 0.05) (Figure 3B,C). To evaluate the impact of miR-425-5p on the value-added efficiency of BMEC, we conducted EDU assays. EDU detection revealed a significantly higher percentage of positive cells in the miR-425-5p mimic group compared to those in the NC group, while the miR-425-5p inhibitor group showed a significantly lower percentage of positive cells compared to those in the INC group (*p* < 0.05) (Figure 3D–F). Subsequently, to validate the accuracy of the phenotyping test, we assessed the expression levels of value-added-related genes in BMEC within each experimental group following transfection. RT-qPCR analysis revealed a significant upregulation of PCNA, CDK2, MCM3, and CDK4 expression in the miR-425-5p mimic group compared to the NC group and a significant downregulation in the miR-425-5p inhibitor group compared to that in the INC group (*p* < 0.05) (Figure 3G–J). The protein expression levels of PCNA and CDK4 were validated. The results demonstrated a significant upregulation in the expression levels of PCNA and CDK4 in the miR-425-5p mimic group, whereas a significant downregulation was observed in the miR-425-5p inhibitor group compared to the NC group (*p* < 0.05) (Figure 3K–M).

### 3.4. miR-425-5p Regulates BMECs by Targeting TOB2

**Figure 4:**
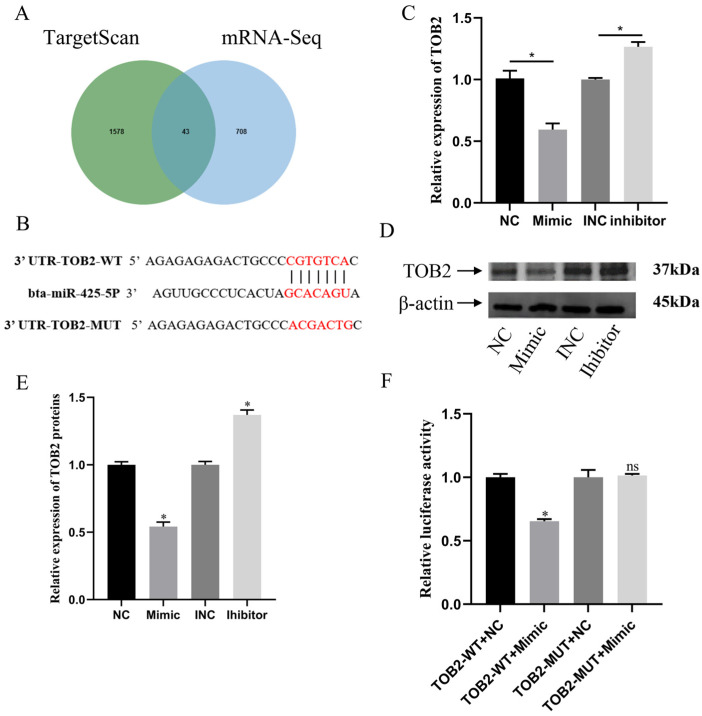
*TOB2* was identified as a direct target of miR-425-5p. (**A**) The Venn diagram illustrates the overlap between predicted target genes of miR-425-5p according to TargetScan and downregulated genes identified through mRNA-seq analysis (padj < 0.05, log2foldchange < 0). (**B**) Sequence alignment of the binding site for the *TOB2* gene with miR-425-5p targets. (**C**) The changes in *TOB2* gene expression levels in BMEC were validated through RT-qPCR after the overexpression and suppression of miR-425-5p expression (*n* = 9). (**D**,**E**) Alterations in the expression levels of *TOB2* protein were assessed following overexpression and inhibition of miR-425-5p, with β-actin serving as an internal reference (*n* = 3). (**F**) Luciferase reporter assay was conducted to investigate the binding of *TOB2* gene with miR-425-5p target (*n* = 9). *β-actin* was used as an internal reference. The data are presented as means ± SEM. * *p* < 0.05, ns (no significance).

To further investigate the potential mechanisms underlying the miR-425-5p-mediated regulation of BMEC proliferation, we employed TargetScan to predict the putative target genes of miR-425-5p. Among a total of 1621 genes, we identified 20 candidate genes associated with cell cycle regulation, including the 3′-UTR region of TOB2 (Figure 4A,B). RT-qPCR analysis revealed a significant downregulation of TOB2 expression in the miR-425-5p mimic group, whereas in the miR-425-5p inhibitor group, TOB2 expression levels were significantly upregulated (*p* < 0.05) (Figure 4C). Western blotting experiments revealed a significant reduction in TOB2 protein expression levels in the miR-425-5p mimic group compared to those in the NC group, while there was a significant increase in the miR-425-5p inhibitor group (*p* < 0.05) (Figure 4D,E). To more accuracy validate the targeting relationship between TOB2 and miR-425-5p, we conducted a dual luciferase reporter gene assay. Results showed that the binding of miR-425-5p to the wild-type 3′-UTR of TOB2 mRNA significantly reduced the luciferase reporter gene activity (*p* < 0.05), whereas no significant change was observed in the mutant group (Figure 4F). Collectively, these findings identify TOB2 as a direct target of miR-425-5p.

### 3.5. TOB2 Attenuated the Proliferation Effect of miR-425-5p on BMECs

**Figure 5:**
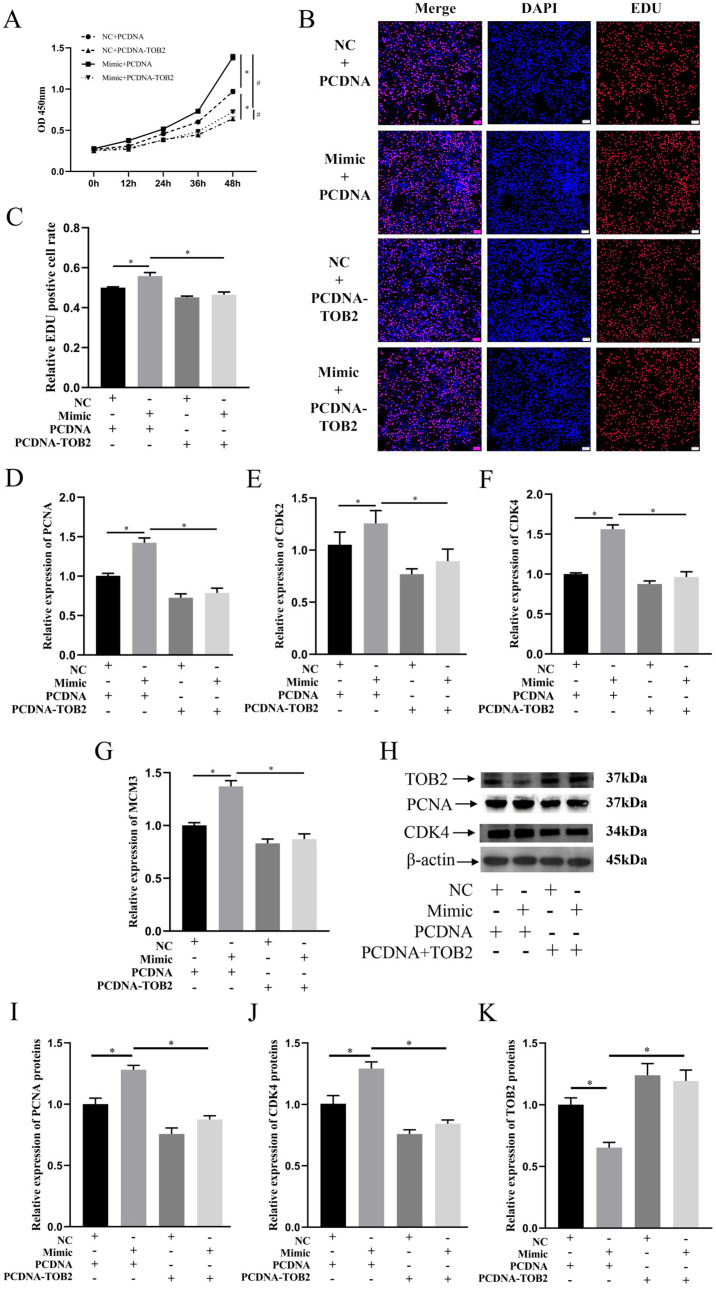
The promotion of BMEC proliferation was inhibited by *TOB2*, indicating its involvement in the regulation of BMEC through suppressing miR-425-5p. (**A**) The viability of BMEC in each group after co-transfection was detected using CCK-8 reagent (*n* = 5). * *p* < 0.05 indicates difference from NC+PCDNA group; # *p* < 0.05 indicates difference from Mimic+PCDNA-TOB2 group. (**B**,**C**) The proportion of value-added cells in BMEC of each group after cotransfection was detected using EDU reagent (*n* = 5). (**D**–**G**) Changes in the expression levels of value-added-related genes (including PCNA, CDK2, CDK4, and MCM3) were detected in each group using RT-qPCR. (**H**–**K**) Changes in protein expression levels of CDK4, PCNA, and TOB2 after co-transfection was detected using Western blotting and visualised as grey scale values (*n* = 3). NC indicates miR-425-5p NC; Mimic indicates miR-425-5p mimic; PCDNA indicates an empty plasmid without any gene inserted; and PCDNA-TOB2 indicates a plasmid with the TOB2 target gene inserted. *β-actin* was used as an internal reference. The data are presented as means ± SEM. * *p* < 0.05.

To elucidate the role of *TOB2* in the regulation of BMEC proliferation mediated by miR-425-5p, BMECs were co-transfected with miR-425-5p mimic or NC together with PCDNA or PCDNA-TOB2 plasmids. CCK-8 results showed that the absorbance at 450 nm significantly increased in the co-transfection group of miR-425-5p mimic and PCDNA compared to the miR-NC + PCDNA group, indicating that the overexpression of miR-425-5p enhanced the proliferative capacity of BMECs. However, when *TOB2* was overexpressed, the promoting effect of miR-425-5p on BMEC proliferation was strongly inhibited (*p* < 0.05) (Figure 5A). EDU detection results further supported this conclusion (*p* < 0.05) (Figure 5B,C). After co-transfection, the RT-qPCR results revealed a significant upregulation in the expression of proliferation-related genes including *PCNA*, *CDK2*, *CDK4*, and *MCM3* upon the overexpression of miR-425-5p. Conversely, when miR-425-5p mimic and PCDNA-TOB2 were co-transfected, there was a notable downregulation observed in the expression of these genes (*p* < 0.05) (Figure 5D–G). Western blotting results showed that the overexpression of miR-425-5p increased the expression levels of proliferation-related gene proteins, while *TOB2* inhibited this phenomenon (*p* < 0.05) (Figure 5H). These results suggest that *TOB2*, as a target gene, inhibits the proliferative promotion of BMECs by miR-425-5p.

## 4. Discussion

Livestock were mostly homeothermic animals that require a stable body temperature. However, the recent high-temperature environments have led to heat stress reactions in these animals, which have severely impacted the breeding industry [32]. Heat stress conditions significantly reduce the reproduction, immune defence, and the production functions of livestock. Heat stress can have a significant impact on milk quality in cows, resulting in decreased production and lower protein and fat content. In severe cases, it can even lead to acute mastitis and mortality. This decrease in milk production was due to reduced feed intake as cows regulate their heat balance by decreasing the heat generated from digesting food. Alternatively, it was believed by some scholars that heat stress can impact the development of BMECs, resulting in a reduction in milk production [33]. Therefore, it was crucial to improve the inhibitory effect of heat stress on BMEC development to enhance milk production in cows in hot environments.

The presence of heat shock proteins (HSPs) was ubiquitous across a wide range of organisms, with genomes from bacteria to humans containing genes encoding these small molecular chaperones. Furthermore, the expression levels of HSPs were closely associated with thermal stimulation [34]. During our investigation, we observed a gradual increase in the expression of HSF1 within BMEC as the duration of heat stimulation was extended. Our findings reveal that miR-425-5p was expressed at higher levels in mammary tissue compared to other tissues due to the pronounced tissue specificity of miRNAs. Under conditions of heat stress, the expression levels of miR-425-5p within BMECs were significantly reduced. Therefore, we investigated the function of miR-425-5p in BMECs. Previous studies have shown that miR-425-5p regulates the proliferation, differentiation, and migration of cancer cells by targeting various genes [22,23,25]. The miR-425-5p may potentially exert a pivotal role in mitigating the inhibition of cell proliferation induced by heat stress stimulation.

The analysis of mRNA-seq data resulted in 753 differentially expressed genes (DEGs), including both upregulated and downregulated transcripts. Some of these genes have been linked to cellular value-added processes [35,36,37]. The categorization of these DEGs was facilitated by the subsequent GO and KEGG analyses. The analysis of GO enrichment revealed a high number of DEGs involved in cellular amide metabolic process, the regulation of protein metabolic process, amide biosynthetic process, and the regulation of cellular protein metabolic process, suggesting a strong association with protein metabolism.

KEGG pathway enrichment analysis revealed a significant representation of pathways associated with enhancing cellular viability, suggesting a possible regulatory role for miR-425-5p in enhancing the viability of BMEC under HS conditions. Additionally, a significant enrichment of DEGs was observed in KEGG pathways critical for thermogenesis, implicating genes such as *NDUFB9*, *ATP6*, and *COX4I1*. These genes were the integral components of the mitochondrial oxidative respiratory chain, highlighting their involvement in the metabolic response to thermal challenges [38,39,40]. The convergence of these findings suggests a comprehensive gene regulatory network, potentially coordinated by miR-425-5p, that may influence the cellular capacity for proliferation addition and thermogenesis in BMEC.

To investigate the impact of miR-425-5p on the proliferation characteristics of BMEC, we used CCK-8 and EDU assays to evaluate cell viability and proliferation capacity. Our results showed a significant enhancement in the proliferation capacity of BMEC transfected with the miR-425-5p mimic group compared to the BMEC transfected with the NC group. In contrast, the group that received the miR-425-5p inhibitor showed a significant decrease in value-added capacity. Similar results were obtained from studies involving cancer cells, further confirming the role of miR-425-5p in promoting the proliferation properties of ovarian and lung cancer cells [23,41].

In addition, to evaluate cellular proliferation processes, it was important to examine the expression levels of relevant genes. *PCNA* and *MCM* were crucial regulators involved in DNA replication and repair, and their presence plays a pivotal role in determining cellular proliferation processes. Therefore, *PCNA* and *MCM* were commonly used as markers to assess cellular proliferation processes [42,43]. The expression of *CDK2* and *CDK4*, two cell cycle proteins, enables the progression into the S-phase and facilitates cellular value-addition [44,45]. RT-qPCR analysis conducted on transfected BMEC consistently indicated a significant upregulation of value-added gene expression in the miR-425-5p mimic group, in contrast to the NC group. Conversely, a significant downregulation of value-added gene expression was observed in the miR-425-5p inhibitor group. The Western blotting assay results further substantiated these findings, demonstrating a parallel correspondence.

To gain a deeper understanding of the mechanism underlying the action of miR-425-5p, we integrated differentially expressed genes (DEGs) identified through mRNA-seq analysis (padj < 0.05, log2foldchange < 0) with genes predicted by TargetScan Human 8.0. Among the overlapping genes, *TOB2* emerged as a potential target of miR-425-5p. This relationship was further validated using molecular assays, confirming *TOB2* as a direct target of miR-425-5p. Previous studies have implicated *TOB2* in the regulation of cellular cycle [21,46]. *TOB2* belongs to the *TOB* anti-proliferative protein family, exerting its influence on cell cycle progression and primarily inhibiting the transition from the G0/G1 phase to the S phase [47]. Chen’s investigation revealed that mutations in the *TOB2-S254D* complex caused the accelerated degradation of transcripts encoding cell cycle-related proteins, resulting in an antiproliferative effect [48]. Furthermore, *TOB* interacts with *CPEB* and *Caf1* to form the ternary complex *CPEB-Tob–Caf1*, which negatively regulates the expression of *c-myc* by accelerating the de-adenylation and decay of *c-myc* mRNA. However, the expression of the *c-myc* gene was crucial for cell growth; thus, TOB indirectly controls cellular proliferation [49].

To verify the impact of miR-425-5p on value-addition in BMEC through its influence on *TOB2*, co-transfection experiments were performed involving NC, miR-425-5p mimic, PCDNA, and PCDNA-TOB2 vectors in BMEC. The subsequent assessments using CCK-8, EDU, RT-qPCR, and Western blotting assays revealed that *TOB2* counteracted the enhancing effect of miR-425-5p on BMEC value-addition. These findings suggest that miR-425-5p promotes value-addition in BMECs by specifically targeting and regulating *TOB2*. Previous investigations have also shown that miR-425-5p regulates cellular value-addition through its targeting of *AFF4* and *ANXA2* [23,50].

## 5. Conclusions

The findings from this research demonstrate that HS leads to a decrease in miR-425-5p expression, and increasing its expression level can mitigate the inhibitory effect of HS on the proliferation process in BMEC. Furthermore, we have identified *TOB2* as a target of miR-425-5p, which co-regulates the proliferation process in BMEC. These results not only lay a foundation for further comprehensive investigations into the functional role of miR-425-5p in BMEC but also present new potential targets to alleviate the inhibitory effects of HS on BMEC value-addition.

## Data Availability

The mRNA-seq datasets generated during the current study are available in NCBI SRA (PRJNA1055257). Data are contained within the article.

## Supplementary Materials

The following supporting information can be downloaded at: https://www.mdpi.com/article/10.3390/genes15020174/s1, Table S1 shows the sequencing raw data quality and alignment statistics information; Table S2 shows the DEGs between the sequenced mimic group and the NC group; Table S3 shows all entries generated by GO enrichment of DEGs; Table S4 shows all entries generated by KEGG pathway enrichment of DEGs. Table S5 shows the sequence information of miR-425-5p analogs; Table S6 shows the RT-qPCR primer sequences for all genes in this study; Table S7 shows the information of all miR-425-5p target genes predicted by Targetscan.
